# Hydrogen and Water Bonding between Glycosaminoglycans and Phospholipids in the Synovial Fluid: Molecular Dynamics Study

**DOI:** 10.3390/ma12132060

**Published:** 2019-06-27

**Authors:** Piotr Bełdowski, Adam Mazurkiewicz, Tomasz Topoliński, Tomasz Małek

**Affiliations:** 1 Institute for Multiscale Simulation, Cluster of Excellence “Engineering of Advanced Materials”, Friedrich-Alexander-Universität Erlangen-Nürnberg, Cauerstrasse 3, 91058 Erlangen, Germany; 2 Institute of Mathematics and Physics, UTP University of Science and Technology, Kaliskiego 7 Street, 85-796 Bydgoszcz, Poland; 3 Mechanical Engineering Department, UTP University of Science and Technology, Kaliskiego 7 Street, 85-796 Bydgoszcz, Poland; 4 Student Scientific Club “BioMed”, Mechanical Engineering Department, UTP University of Science and Technology, Kaliskiego 7 Street, 85-796 Bydgoszcz, Poland

**Keywords:** synovial fluid, osteoarthritis, hyaluronic acid, chondroitin sulphate, phospholipids, molecular dynamics simulation, hydrogen bond, water bridges

## Abstract

Synovial fluid is a lubricant of the synovial joint that shows remarkable tribological properties. These properties originate in the synergy between its components, with two of its major components, glycosaminoglycans (GAGs) and phospholipids (PLs), playing a major role in boundary and mixed lubrication regimes. All-atom molecular dynamic simulations were performed to investigate the way these components bond. Hyaluronic acid (HA) and chondroitin sulphate (CS) bonding with three types of lipids was tested. The results show that both glycosaminoglycans bind lipids at a similar rate, except for 1,2-d-ipalmitoyl-sn-glycero-3-phosphoethanolamine lipids, which bind to chondroitin at a much higher rate than to hyaluronan. The results suggest that different synovial fluid lipids may play a different role when binding to both hyaluronan and chondroitin sulphate. The presented results may help in understanding a process of lubrication of articular cartilage at a nanoscale level.

## 1. Introduction

The joint surfaces covered with cartilage are separated by a thin layer of joint fluid called synovial fluid (SF). Synovial fluid fulfils a number of functions, the most important of which are a reduction of friction during the mutual movement of joint surfaces, increasing the uniformity of load distribution on cartilage surfaces and nutrition as a result of pressing and squeezing the fluid from its volume under the influence of mechanical loads. Because cartilage does not have its own blood vessel networks, its nutrition is based on the diffusion of nutrients from synovial fluid.

**Figure 1:**
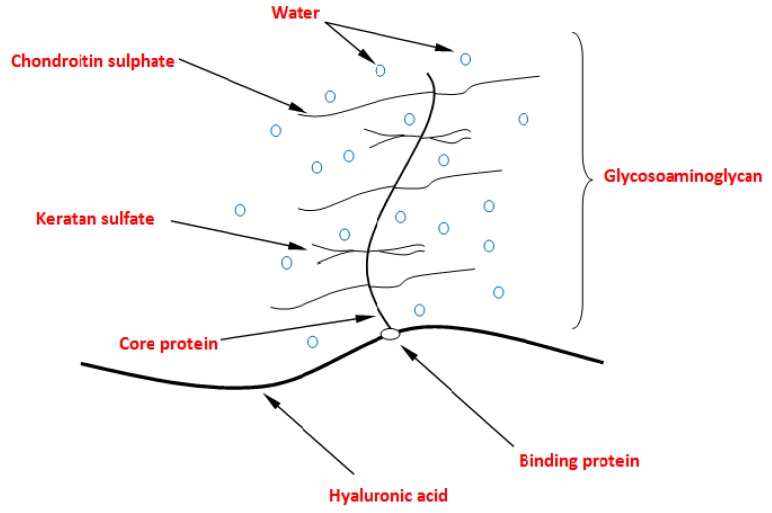
Proteoglycan aggregate (elaborating on [8]).

Articular cartilage is composed of cartilage cells—chondrocytes submerged in the extracellular matrix (ECM) [1]. The main matrix components are collagen fibers, which form a scaffold that gives cartilage shape, cohesion and mechanical properties. The spaces between the fibers are filled with high-molecular proteins and glycosaminoglycan (GAG) complexes called proteoglycans. Proteoglycans, like fibers, form their own spatial structures with a scaffolding character that has the ability to bind large amounts of water [2,3,4,5]. Proteoglycans are responsible for the correct work of cartilage (i.e., appropriate flexibility, and the ability to absorb mechanical loads and transfer them from one bone surface to another). One of the most important proteoglycans (Figure 1) in the joint cartilage matrix is aggrecan, which consists of chains of chondroitin sulphate (CS) and keratan sulphate (KS) that are attached to the protein core. These particles attach to hyaluronic acid (HA) molecules to form a proteoglycan aggregate. This aggregate usually contains more than 100 CS chains and about 20–50 KS chains. Phospholipids (PLs) play an important role in joint lubrication. In the hydrodynamic model of joint lubrication, it is assumed that few bilayers of phospholipids are formed in the joint, with a total thickness per bilayer of about 5 nm [6,7]. Phospholipids are organized in the layer as a result of electrostatic forces between the phospholipid head and cartilage and their polarization.

The bilayers consist of two oppositely arranged layers of lipid molecules with hydrophobic hydrocarbon ends facing the center of the layer, with polar hydrophilic phosphate groups on the outside [6]. During the movement in the pond, both layers slide across each other, which reduces friction and, as a consequence, resistance to movement.

Synovial fluid is a dialysate of blood plasma. Its pH = 7.2–7.4 [9]. The protein content does not exceed 21 g/L, and its cholesterol does not exceed 70 g/L [10]. The amount of glucose and urea electrolytes is comparable to the concentration found in blood serum. The joint fluid contains HA, CS and KS. HA contains recurrent disaccharide units composed of N-acetylglucosamine and glucuronic acid, CS contains N-acetylgalactosamine and glucuronic acid and KS contains N-acetylglucosamine and galactose. 

**Figure 2:**
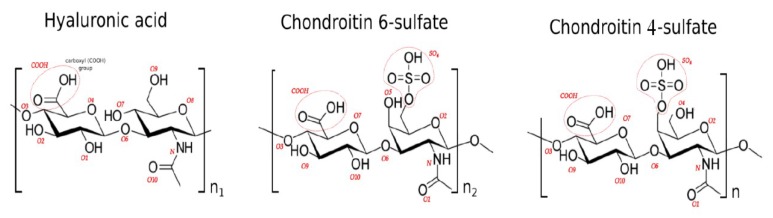
Chemical structures of the units in hyaluronic acid (HA), chondroitin 6-sulphate (CS-6) chains and chondroitin 4-sulphate (CS-4) chains.

The aim of this work is to present the interactions between phospholipids (PLs) and glycosaminoglycans (GAGs) in terms of direct (hydrogen) and indirect (water bridge) bonds. Namely, between lipids: (i) dipalmitoyl phosphatidylcholine (DPPC) (40% of all lipids in synovial fluid), (ii) 1,2-Dipalmitoyl-sn-glycero-3-phosphoethanolamine (DPPE) (30% of all lipids in synovial fluid) and (iii) sphingomyelin (SPH) (30% of all lipids in synovial fluid) and HA/(CS-4 and CS-6) (Figure 2). We seek to understand how particular GAGs can promote aggregation of PLs in the synovial fluid milieu. 

## 2. Materials and Methods 

**Figure 3:**
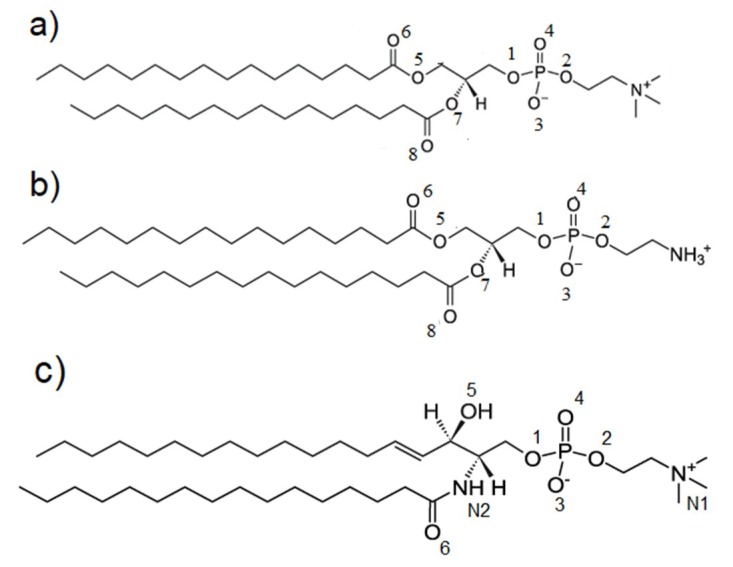
Chemical structures of the units in phospholipids (PLs): (**a**) DPPC, (**b**) DPPE and (**c**) SPH. Abbreviations has been explained above. The oxygen and nitrogen atoms are numbered to present a contact maps, see Figures 9 and 10.

HA, CS-4 and CS-6 structure modification with YASARA Structure software (Vienna, Austria) [11] is described in [12]. Three types of phospholipids—DPPC, DPPE, and SPH (Figure 3)—were used to look at their interactions with the abovementioned glycosaminoglycans. Molecules were placed randomly in a simulation box that was followed by system minimization with a time step of 1 fs. Next, water molecules were added, followed with another 1000-step minimization with a 1-fs time step. The total number of atoms in all the cases was ~150,000, including water molecules (4400 of GAGs, and 7500 of PL). Isothermal–isobaric ensemble all-atom simulations were performed under the following conditions: temperature 310 K, pH = 7.0 in 0.9% NaCl (0.154 M) aqueous solution (a four-site (TIP3P) model of water [13]), with a timestep of 2 fs. Berendsen barostat [14] with a relaxation time of 1 fs were used to maintain constant temperature and pressure. The final concentrations of HA and CS were the same, C_CS_ = C_HA_= 5·10^−7^ M, and both molecules were of the same weight—40 kDa. Concentrations used were chosen to enable PL molecules to bind to GAGs in a relatively short time, and thus they do not refer to any value taken from the literature.

### 2.1. Molecular Dynamics Force Field

AMBER03 all-atom force field [15] was applied to perform simulations of hyaluronan, chondroitin 4-, chondroitin 6- and phospholipid molecules in the aqueous solution. The AMBER03 potential function describing interactions among particles takes into account electrostatic, van der Waals, bond, bond angle and dihedral terms, and was previously described in [12].

### 2.2. Hydrogen Bond Identification

We utilized the YASARA definition of a hydrogen bond (H-bond), where an H-bond is considered formed when the hydrogen bond energy is greater than 6.25 kJ/mol, which is 25% of the optimum value of 25 kJ/mol. The following formula yields the bond energy in kJ/mol as a function of the hydrogen–acceptor distance and two scaling factors:

(1) $$E_{HB}=25\cdot \frac{2.6-max\left(Dis_{H-A,}2.1\right)}{0.5}\cdot Scale_{D-A-H}\cdot Scale_{H-A-X}$$

where the first scaling factor depends on the angle formed by donor–hydrogen–acceptor, and the second scaling factor is derived from the angle formed by hydrogen–acceptor–X, where the X stands for the atom covalently bound to the acceptor. 

Both scaling factors vary from 0 to 1 as follows:

(2) $$Scale_{H-A-X}\{\begin{array}{c} 0\\ \frac{\alpha -100}{165-100}\\ 1 \end{array}\begin{array}{c} in\;range\;0..100\;degrees\\ \;\;in\;range\;100..165\;degrees\\ in\;range\;165..180\;degrees \end{array}$$

if X is a heavy atom, the second scaling factor is

(3) $$Scale_{H-A-X}\{\begin{array}{c} 0\\ \frac{\alpha -85}{95-85}\\ 1 \end{array}\begin{array}{c} in\;range\;0..85\;degrees\\ in\;range\;85..95\;degrees\\ in\;range\;95..180\;degrees \end{array}$$

if X is a hydrogen atom, slightly smaller angles are allowed, and the scaling factor is then

(4) $$Scale_{H-A-X}\{\begin{array}{c} 0\\ \frac{\alpha -75}{85-75}\\ 1 \end{array}\begin{array}{c} in\;range\;0..75\;degrees\\ in\;range\;75..85\;degrees\\ in\;range\;85..180\;degrees \end{array}$$

where α is an angle between three chosen atoms. If the acceptor forms more than one hydrogen bond, the one with the lowest scaling factor is taken. All scaling factors in their middle cases are linear functions [16].

Solvent accessible surface as evaluated in this study consists of all the points that the center of the water probe (i.e., the nucleus of the oxygen atom in the water molecule) can reach while rolling over the solute, nd the procedure for calculating this variable is been presented in [17,18].

## 3. Results

**Figure 4:**
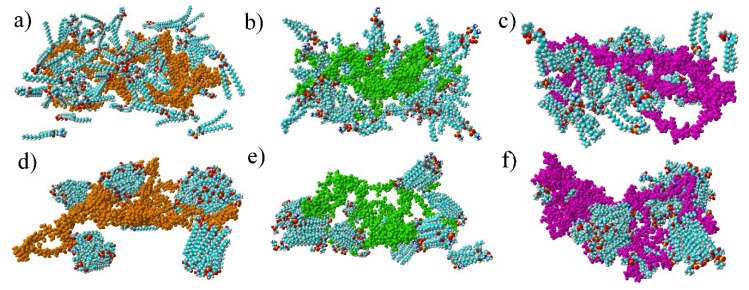
Structures of simulated systems at the beginning and after 20 ns: (**a**) HA + DPPC initial form and (**d**) HA + DPPC final form; (**b**) CS-6 + DPPE initial form and (**e**) CS-6 + DPPE final form; (**c**) CS-4 + SPH initial form and (**f**) CS-4 + SPH final form. HA has been colored orange, CS-6 green and CS-4 pink. Colors of lipids are: turquoise (carbon), white (hydrogen), red (oxygen), yellow (phosphorus) and blue (nitrogen).

**Figure 5:**
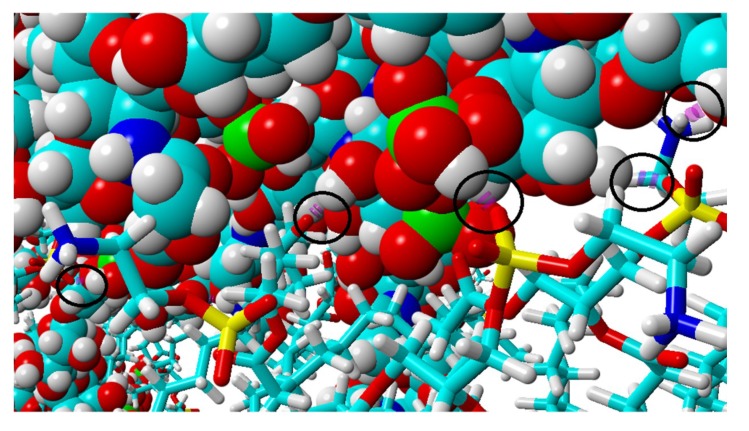
Visualization of H-bonds marked with black circles and pink dotted lines. The picture shows bonding between CS-4 and DPPE. For better visualization, DPPE takes the form of sticks, whereas CS-4 is represented as balls. Colors of atoms are: turquoise (carbon), white (hydrogen), red (oxygen), yellow (phosphorus), blue (nitrogen) and green (sulfur).

**Figure 6:**
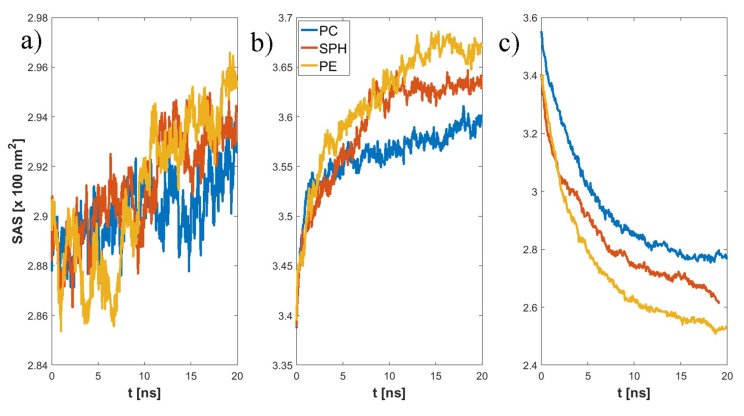
Time evolution of solvent accessible surface: (**a**) HA, (**b**) CS-6, (**c**) CS-4.

**Figure 7:**
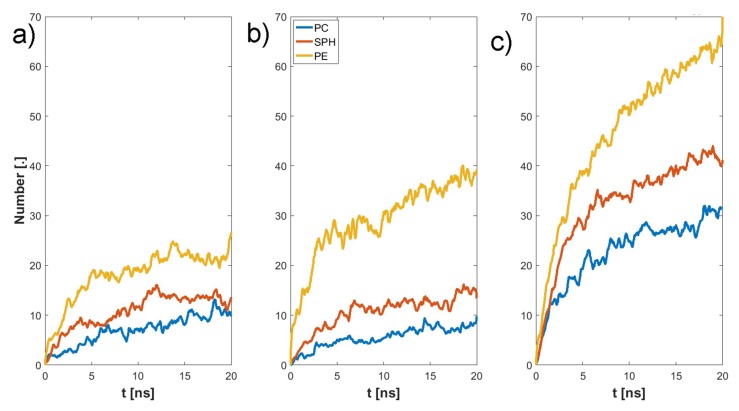
Time evolution of the number intermolecular of GAG–PL H-bonds: (**a**) HA, (**b**) CS-6, (**c**) CS-4.

**Figure 8:**
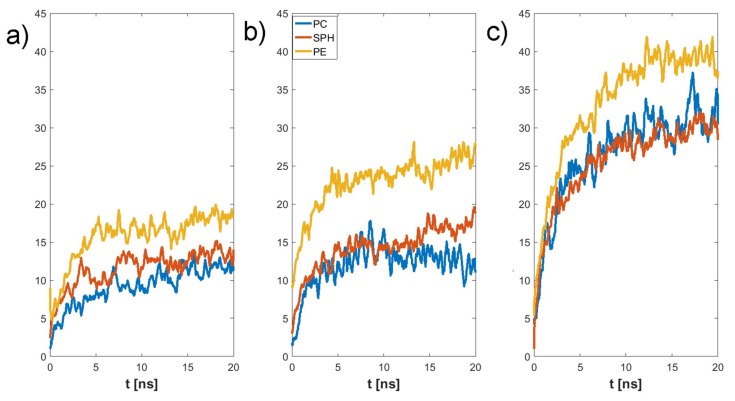
Time evolution of the number of intermolecular GAG–PL water bridges: (**a**) HA, (**b**) CS-6, (**c**) CS-4.

The final structure of the two chosen examples has been presented in Figure 4. Specific H-bonds between chosen GAGs and PLs have been presented in Figure 5. Solvent accessible surface (SAS) is a useful variable in showing the structure dynamics of proteins, biopolymers, and so on [15,19]. The changes of GAG SASs are shown in Figure 6. The difference in behavior of CS-4 can be a result of a much higher affiliation with PLs. Namely, PLs in this case create more compact structures with CS-4, disabling water from penetrating its network more deeply. All phospholipids present in the system were adsorbed at the GAG surfaces. Figure 7 presents the time evolution for a number of intermolecular H-bonds. Among all cases, only HA-DPPC and CS-DPPE systems did not reach the plateau, but as there are significant differences between all systems we were more interested in the H-bond distribution rather than a steady state, as the differences can be insignificant. On the other hand, the total number of intermolecular water bridges reached the plateau after ~10 ns, as one can see in Figure 8.

**Figure 9:**
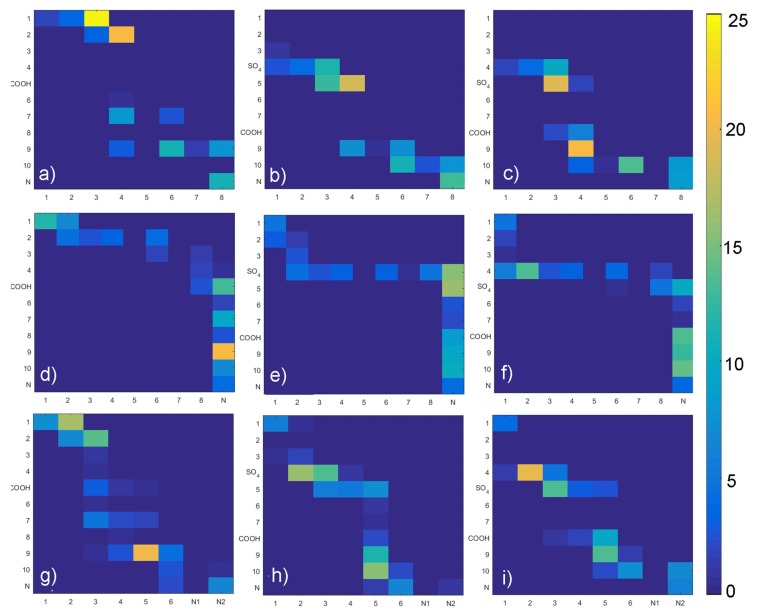
Intermolecular GAG–PL H-bonds map: (**a**) HA-DPPC, (**b**) CS-6-DPPC, (**c**) CS-4-DPPC, (**d**) HA-DPPE, (**e**) CS-6-DPPE, (**f**) CS-4-DPPE, (**g**) HA-SPH, (**h**) CS-6-SPH, (**i**) CS-4-SPH. Legends show a percentage for each pair as compared with the total number of H-bonds formed.

**Figure 10:**
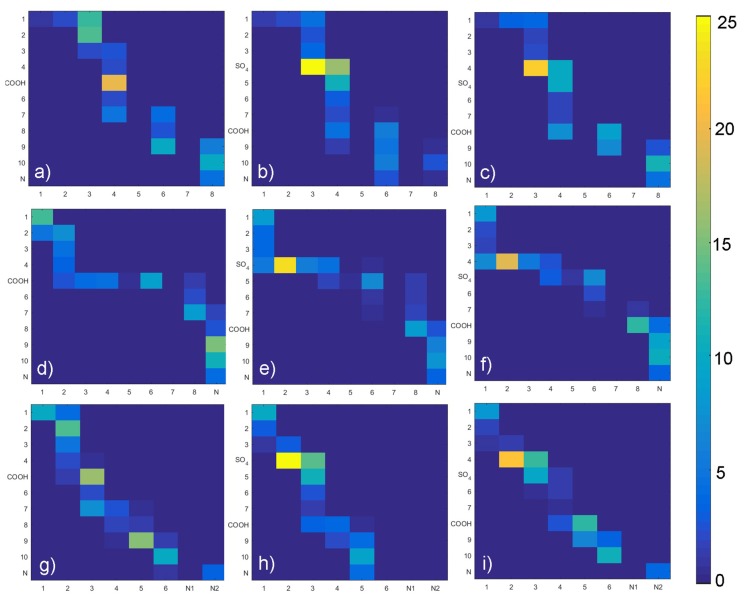
GAG–PL water bridges map: (**a**) HA-DPPC, (**b**) CS-6-DPPC, (**c**) CS-4-DPPC, (**d**) HA-DPPE, (**e**) CS-6-DPPE, (**f**) CS-4-DPPE, (**g**) HA-SPH, (**h**) CS-6-SPH, (**i**) CS-4-SPH. Legends show a percentage for each pair as compared with the total number of water bridges formed.

**Figure 11:**
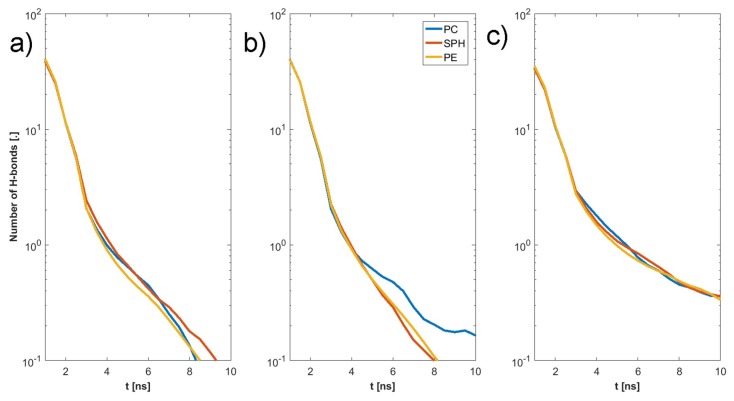
Intermolecular H-bond duration GAG–PLs: (**a**) HA, (**b**) CS-6, (**c**) CS-4.

**Figure 12:**
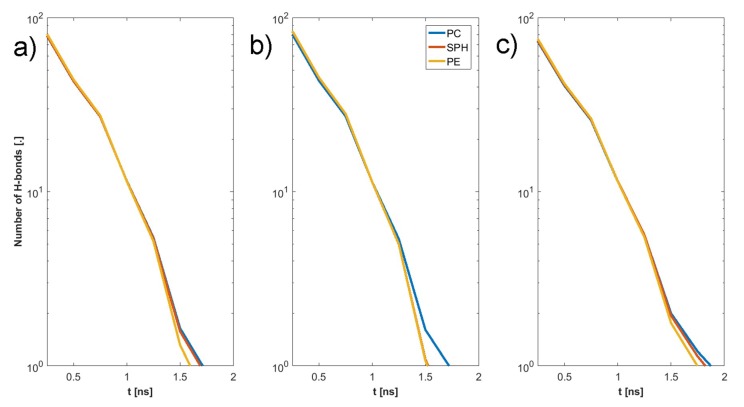
Intermolecular water bridge duration GAG–PLs: (**a**) HA, (**b**) CS-6, (**c**) CS-4.

In the next step we have listed all H-bond pairs between (i) PLs and GAGs, and (ii) water and other molecules. From the water-involved H-bonds, we have selected those that, at an analyzed time step, connected to those that bound to both molecules, which we call a “water bridge”. If a selected pair appeared in the simulation more than once, it was treated as a single hydrogen bond. However, we counted the number of particular pairs to estimate the H-bond duration. The results of this analysis have been presented in Figure 9, Figure 10, Figure 11 and Figure 12. Figure 9 shows that there is a difference of bonding sites for both GAGs depending on the PLs used. The difference in H-bond duration presented in Figure 11 show that there CS-4 exhibit longer lasting bonding with PLs. Water bonding duration, see Figure 12, show that there is no significant changes between cases.

## 4. Discussion

Disturbances in joint surface lubrication cause the friction coefficient between joint surfaces to increase, resulting in further damage of the cartilage. This leads to various diseases (e.g., osteoarthritis). It is therefore necessary to know the processes occurring between the individual components of this fluid that affect its properties.

This study seeks to present a difference in affinity between the chosen GAGs and phospholipids present in the synovial fluid system. Our results give more molecular details to interactions, as we present the mechanism of H-bond and water bridge creation between specific sites inside and between molecules. 

The synergy in the SF system has been shown to be the key to understanding the remarkable properties of SF [20,21,22]. HA is the major component of SF and the most important molecule regarding its shock-absorbing properties [9]. Thus, HA interactions with PLs are of importance [23,24]. Osteoarthritis is connected with an increased concentration of PLs, as well as a change in the polydispersity of HA [25,26,27,28,29,30,31]. Figure 7 and Figure 8 show that HA s less reactive in bonding PL than CS. This is a result of the presence of SO_4_ group in CS that promote the formation of more-direct H-bonds and the vast majority of water bridges. This will likely influence the stiffness of both chains, as there is a limited number of H-bonds that a single atom can create. Figure 9 show that DPPC lipid can only bind only to five sites in both HA and CS. On the other hand DPPE can bind to all sites in both molecules. Besides being an additional factor, the creation of intermolecular water bridge binding can change a local organization of solvents, thus modifying GAG properties (presented in Figure 10). However, the intermolecular water bridges would have a smaller effect, as they are shorter than direct H-bonds, as presented in Figure 11 and Figure 12, respectively. The intramolecular H-bonds inside HA, as well as the creation of water bridges inside its molecule, increase its persistence length. Thus, the presence of more complex PL aggregates can highly affect its stiffness. On the other hand, PLs themselves can replace the stiffness of water supporting GAGs. 

Our future work will focus on presenting a change in persistence length of GAGs as affected by the presence of PLs. In this study, our goal was to present how both molecular species bind with one another depending on the type of both lipid and GAG used.
